# Survival of bovine-associated serotypes of *Salmonella enterica* in bedding sand

**DOI:** 10.3168/jdsc.2022-0305

**Published:** 2022-12-22

**Authors:** Hannah Pilch, Nicole Aulik, Donald Sockett, Charles J. Czuprynski

**Affiliations:** 1 Department of Pathobiological Sciences, University of Wisconsin-Madison 53706; 2 Wisconsin Veterinary Diagnostic Laboratory, School of Veterinary Medicine, University of Wisconsin-Madison 53706; 3 Food Research Institute, College of Agricultural and Life Sciences, University of Wisconsin-Madison 53706

## Abstract

Cattle persistently infected with bovine-adapted serotypes of *Salmonella enterica* are an important animal health and food safety issue. One possible mechanism by which infection is sustained in a dairy herd is by survival of *Salmonella* in sand used as bedding material. In this study we assessed the survival of 10^7^ to 10^8^ cfu bovine-associated serotypes of *Salmonella enterica* (sv. Cerro, Dublin, and Heidelberg) in sterile sand, recycled bedding sand, and gray water collected from a Wisconsin dairy farm. All 3 serotypes persisted at relatively high numbers (>10^6^ cfu/g) for at least 28 d in sterile sand, with *Salmonella* sv. Dublin decreasing less than 1 log_10_ over 70 d. To our surprise, when low numbers of *Salmonella* sv. Dublin (10^3^ cfu) were inoculated into sterile sand, the organism multiplied within 3 d to approximately 10^6^ cfu/g sand and persisted at that level for 28 d. When we inoculated *Salmonella* sv. Dublin into recycled bedding sand or sand taken directly from cow pens, we observed a significant decrease in colony-forming units by d 7. In contrast, we observed a significant increase in colony-forming units when *Salmonella* sv. Dublin was inoculated into gray water from the sand recycling system. These data demonstrate that *Salmonella* can persist for extended periods of time in bedding sand, although this is limited to some extent by the native microbiota in recycled bedding sand.

## Highlights

• Bovine-associated serotypes of *Salmonella enterica* (sv. Cerro, Dublin, and Heidelberg) persisted at relatively high numbers for at least 28 days after inoculation in sterile sand.
• When *Salmonella* sv. Dublin was inoculated into recycled bedding sand or sand taken directly from cow pens, there was a significant decrease in cfu by day 7.
• There was a significant increase in cfu when *Salmonella* sv. Dublin was inoculated into gray water from the sand recycling system.
• *Salmonella* can persist for extended periods of time in bedding sand, although this is limited to some extent by the native microbiota in recycled bedding sand.

## Graphical Abstract

**Figure:**
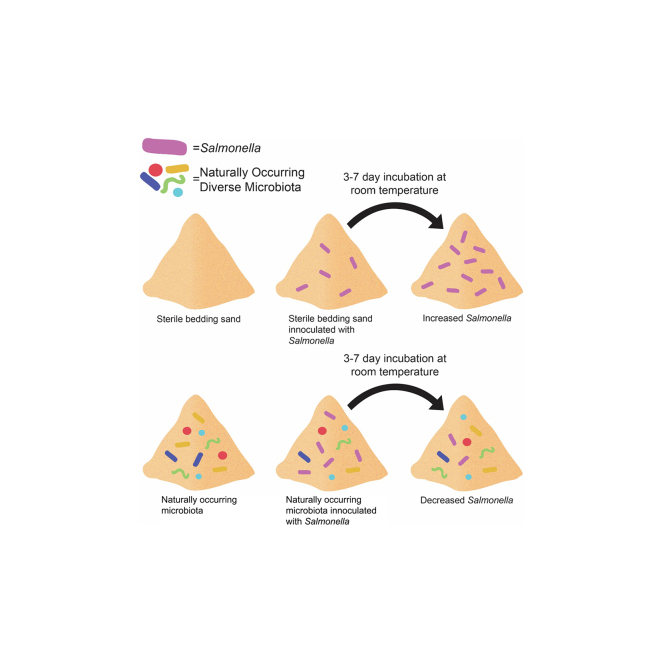
**Summary:***Salmonella enterica* sv. Dublin inoculated into sterile bedding sand can survive and proliferate when incubated at room temperature for up to 7 days. However, inoculation of *Salmonella enterica* sv. Dublin into bedding sand that contains a naturally occurring microbiota results in a decrease in cfu of *Salmonella enterica* sv. Dublin under the same conditions.

*Salmonella* serotypes such as *Salmonella enterica* sv. Typhimurium and *Salmonella enterica* sv. Dublin (Wray and Davies, 2000) are a common cause of enteric disease in young calves (2 to 4 wk of age). Host-adapted serotypes like *Salmonella enterica* sv. Dublin are also problematic in adult cattle, which can experience pyrexia, lethargy, diarrhea, and in some cases abortion (McGuirk and Peek, 2003). Adult cows can become asymptomatic carriers that intermittently shed *Salmonella* sv. Dublin in their feces for life, making it difficult to eradicate *Salmonella* from a herd (Veling et al., 2000; Wray and Davies, 2000). In addition to being a significant concern for cow health, *Salmonella* is also a major human foodborne disease pathogen that is frequently associated with beef and dairy products (Arthur et al., 2008; Centers for Disease Control and Prevention, 2022).

Although the natural reservoir of *Salmonella* is the gastrointestinal tract, it can survive in environmental reservoirs from which it can infect new animals. Studies have shown *Salmonella* can survive up to 60 d in cow manure held at 20°C, and up to 152 d in sterilized well water (Wray and Sojka, 1981; Himathongkham et al., 1999). Millemann et al. (2000) tracked *Salmonella* in a herd during transport to a slaughterhouse, in the environment, and in the final beef product. These authors isolated the same *Salmonella* clone from all locations sampled, suggesting *Salmonella* persistence in the environment can result in infected cattle and contaminated beef. Clegg et al. (1983) investigated a dairy herd experiencing recurrent outbreaks of *Salmonella enterica* sv. Newport and found the organism persisted for up to 14 mo in pasture soil where the cows grazed. These observations suggest environmental persistence of *Salmonella* is a factor in perpetuating outbreaks.

One potential source of *Salmonella* on dairy farms is the bedding on which cows are housed. Dairy cow comfort is important and bedding type can affect bovine behavioral health, lameness, welfare, and time spent resting (Norring et al., 2008; Gomez and Cook, 2010). Traditional bedding includes straw, sawdust, wood shavings, rubber mats, and sand. The latter carries a significantly lighter bacterial load than organic bedding and is associated with decreased prevalence of mastitis (Fairchild et al., 1982; van Gastelen et al., 2011; Hogan and Smith, 2012). In an effort to reduce costs and increase sustainability, some farms have implemented recycling systems to wash soiled sand and spray it with a disinfectant before reuse.

Previous studies have shown *Salmonella* can persist in beach sand and in children's playground sand pits (Bolton et al., 1999; Staff et al., 2012). These reports suggest that bedding sand could serve as a reservoir for *Salmonella*, from which it could be a source for continued infection of the herd. We recently performed a 16S analysis of the microbiota in bedding sand from a Wisconsin dairy farm in which we identified a core microbiota of 141 operational taxonomic units, with *Acinetobacter*, *Psychrobacter*, *Corynebacterium*, and *Pseudomonas* in the greatest abundance (Pilch et al., 2021) The purpose of the present study was to examine the ability of bovine-associated *Salmonella* serotypes to survive in sterile sand and in recycled bedding sand with a complex microbiota.

Unused bedding sand was obtained from a farm in central Wisconsin and sterilized by autoclaving at 121°C for 15 min. A series of 50-mL sterile conical plastic tubes were filled with 5 mL of sterile dairy sand and the tubes were separately inoculated with approximately 10^8^ cfu/g bovine-associated *Salmonella* (sv. Cerro, Dublin, or Heidelberg). To prepare the inocula, individual *Salmonella* serotypes were incubated at 37°C in trypticase soy broth for 24 h. The cultures were centrifuged at 795 × *g* for 10 min at 22°C, the supernatant discarded, and the pellet suspended in PBS to achieve a bacterial concentration of 10^8^ cfu/mL. Tubes containing sterile bedding sand were inoculated with 0.5 mL of the *Salmonella* suspension (5 × 10^7^ cfu total), and the tubes were capped and incubated at 25°C. Immediately afterward and at various time points (3, 5, 7, 14, 21, and 28 d) 45 mL of PBS was added to each tube and vortexed for 30 s. The sand was allowed to settle, and samples were removed from the supernatant. These were serially diluted in sterile saline, and 0.1 mL of each dilution was spread onto trypticase soy agar with 5% sheep's blood to determine colony-forming units per gram of sand.

Recycled bedding sand was collected from a southern Wisconsin Jersey cow dairy farm (approximately 1,500 cows and heifers) that used a sand recycling system as described previously (Pilch et al., 2021). In brief, sand was mechanically pushed from the top layer of freestalls into the center aisle of the barn and flushed to a central drain using recycled water (gray water). The sand then traveled through the drain to a central reception pit in a separate building on the farm. After the sand was allowed to settle in the reception pit, organic material was siphoned off and sent for composting. Sand collected in the bottom of the reception pit was removed with a rotating auger, doused with a disinfectant (hypochlorous acid), and then dropped into a large pile (“wet recycled sand”). The recycled sand piles were allowed to partially dry in the recycling room for 11 to 14 d, after which the sand was removed and added to freestalls as needed.

We obtained samples at various points in the sand recycling process. Sites from which samples were collected included wet recycled sand immediately after it completed the washing process, washed sand that was stored in a pile for 4 or 14 d, gray water from the reception pit, and sand removed directly from cow freestalls. Sterile wooden tongue depressors were used to collect five 5-mL samples at each site; these were placed in 50-mL conical tubes to create a composite 25-mL sample. After collection, samples were transported on ice to the laboratory and stored at −80°C until used in an experiment.

For each experiment 5-mL samples of sand or gray water were placed into sterile 50-mL conical tubes and inoculated with *Salmonella* sv. Dublin (obtained from the Wisconsin Veterinary Diagnostic Laboratory and originally isolated from bedding sand at a different farm). To prepare the inoculum *Salmonella* sv. Dublin was inoculated into trypticase soy broth and incubated at 37°C for 24 h. The culture was centrifuged at 795 × *g* for 10 min at 22°C, the supernatant discarded, and the pellet resuspended and diluted in PBS to a final concentration of 10^6^ cfu/mL. We added 0.5 mL of the bacterial suspension to tubes containing sand (or gray water) and incubated at 25°C. Immediately afterward, and at 3 and 7 d, we added 45 mL of PBS to triplicate tubes and agitated the tubes with a vortex mixer. After vortexing, samples (0.1 mL) were removed from each tube and serially diluted in PBS. Samples were removed from each dilution tube (0.1 mL) and plated on xylose lysine deoxycholate (XLD) agar to estimate colony-forming units of *Salmonella* and on trypticase soy agar with 5% sheep's blood (trypticase soy agar) to estimate total aerobic bacteria (cfu/g sand or cfu/mL gray water). At each time point uninoculated bedding sand or gray water was treated as described above to determine total aerobic colony-forming units. All samples were tested in triplicate.

For *Salmonella* persistence studies in sterile sand, a Kruskal-Wallis test was performed followed by the Dunn's test to compare the pair-wise significance from the initial colony-forming units of *Salmonella* (d 0) versus colony-forming units on subsequent days. For experiments in which *Salmonella* sv. Dublin was inoculated into various recycled bedding types and gray water, a repeated measures ANOVA was performed followed by the Tukey test to compare the pair-wise significance of the initial colony-forming units of *Salmonella* (d 0) versus colony-forming units on subsequent days. Statistical significance was set at an α of 0.05 for all analyses.

All 3 serotypes of *Salmonella* survived for extended periods when inoculated at 10^7^ to 10^8^ cfu/g in sterile sand (Figure 1). *Salmonella* sv. Cerro significantly increased (*P* = 0.02) during the first 3 d when incubated at 22°C and remained at a constant level for up to 28 d (Figure 1A). *Salmonella* sv. Heidelberg remained at a steady level until d 21 to 28, at which point it decreased more than 1 log_10_ cfu (Figure 1B). *Salmonella* sv. Dublin persisted at steady levels through d 7 and then slowly decreased 0.5 log_10_ cfu/g by d 28 (Figure 1C), and decreased further (approximately 1 log_10_ cfu/g reduction) by 70 d (Figure 1D).

**Figure 1:**
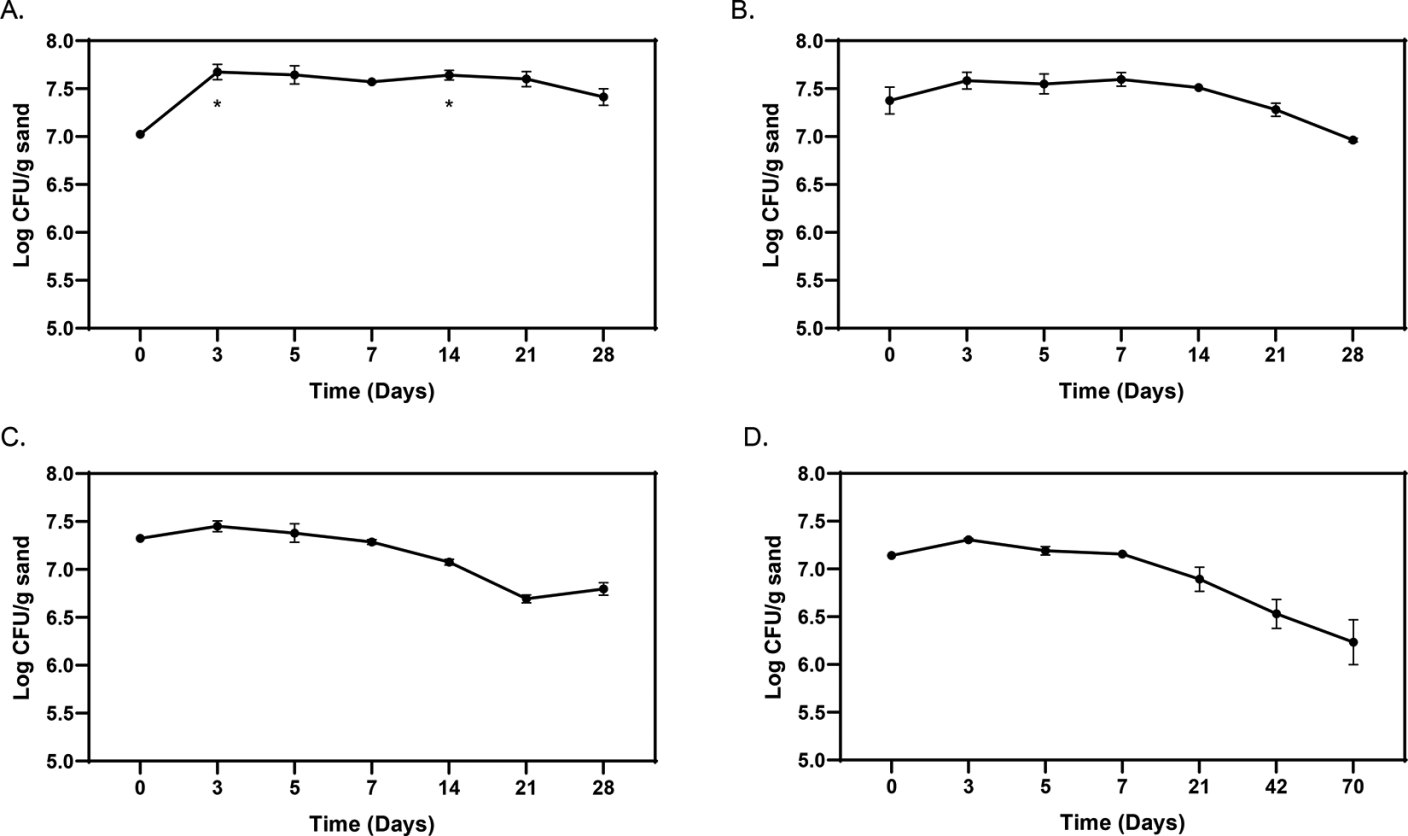
Survival of bovine-associated *Salmonella* serotypes in sterile sand. *Salmonella enterica* sv. Cerro (A), Heidelberg (B), and Dublin (C–D) were grown as indicated herein and inoculated into 5 g of sterile sand in 50-mL conical tubes at starting concentrations of 7.0 to 7.5 log_10_ cfu/g sand. Tubes were incubated at 22°C for up to 28 (A–C) or 70 d (D). Results are expressed as the mean ± SD log_10_ cfu/g. Asterisks (*) represent a significant change in log_10_ cfu/g (*P* < 0.05) from time point 0.

We next examined the effect of inoculum level on survival of *Salmonella* sv. Dublin in sterile bedding sand (Figure 2). When inoculated at approximately 8.0 log_10_ cfu/g there was no significant change in colony-forming units through d 14, followed by a nearly 2 log_10_ decrease (to 6.2 log_10_ cfu/g) by d 28 (*P* = 0.03). In contrast, when inoculated into sand at 5.69 log_10_ cfu/g *Salmonella* sv. Dublin significantly increased approximately 1 log_10_ at d 3 (*P* = 0.02) to 5 (*P* = 0.01) and persisted at 6.48 log_10_ cfu/g through 28 d. To our surprise, sterile sand inoculated with *Salmonella* sv. Dublin at 3.61 log_10_ cfu/g increased 3 log_10_ by d 3, remained significantly increased at 5 (*P* = 0.02) and 14 d (*P* = 0.01), and persisted at 6.37 log_10_ cfu/g through 28 d.

**Figure 2:**
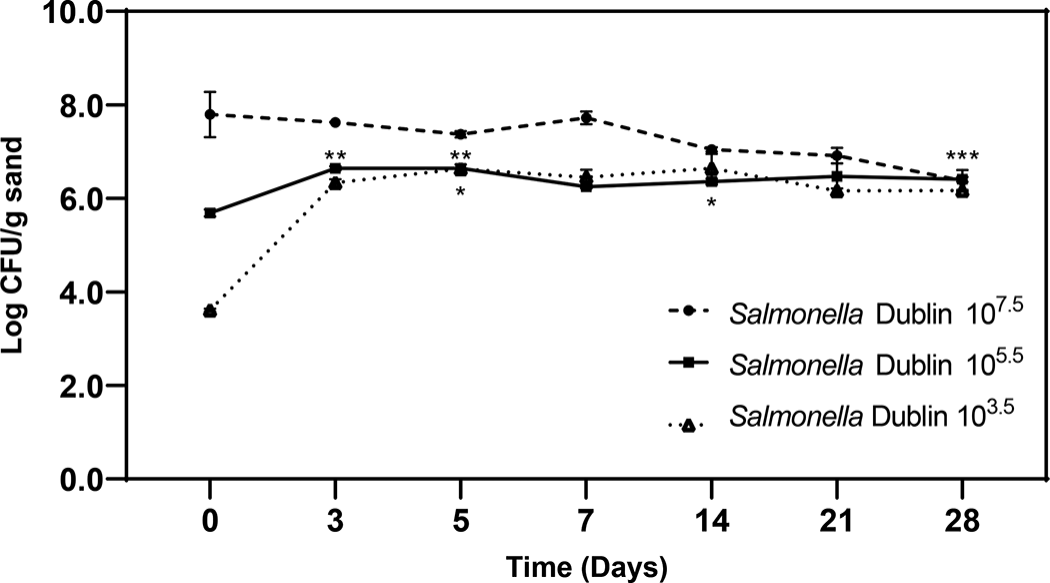
Effect of inoculum on growth and survival of *Salmonella enterica* sv. Dublin in sterile sand. *Salmonella* sv. Dublin was prepared as described in the main text, and various numbers (10^3.5^, 10^5.5^, or 10^7.5^ cfu/g sand) were added to triplicate 50-mL conical tubes containing 5 g of sterile sand. The tubes were incubated at 22°C for up to 28 d. Results are expressed as the mean ± SD log_10_ cfu/g. Asterisks (*, **, and ***) represent a significant change (*P* < 0.05) from time 0 for 10^3.5^, 10^5.5^, or 10^7.5^ cfu/g sand, respectively.

The above results show that several bovine-adapted serotypes of *Salmonella* survive for extended periods of time in sterile sand. However, we acknowledge that a limitation of our study design was that only one strain per *Salmonella* serotype was used in our study. Additional investigation is needed to address whether these results are generalizable to additional isolates of these serotypes, and serotypes not yet tested, of *Salmonella*.

We next asked whether the complex microbiota present in recycled bedding sand would have a detrimental effect on *Salmonella* survival. We observed a significant decrease in colony-forming units on d 3 and 7 (*P* = 0.02; Figure 3A) when *Salmonella* sv. Dublin was inoculated into sand taken directly from freestalls, and after 7 d of incubation when *Salmonella* sv. Dublin was inoculated into recycled sand that had been allowed to dry for 4 d (*P* = 0.02; Figure 3C). There was no significant change in colony-forming units for *Salmonella* sv. Dublin after it was inoculated into freshly recycled sand (Figure 3B), or recycled sand that had been allowed to dry for 14 d (Figure 3D). In contrast, we observed a significant increase (*P* = 0.0001; *P* = 0.0004, respectively) in colony-forming units of *Salmonella* sv. Dublin at 3 and 7 d after inoculation into gray water from the sand recycling system (Figure 3E).

**Figure 3:**
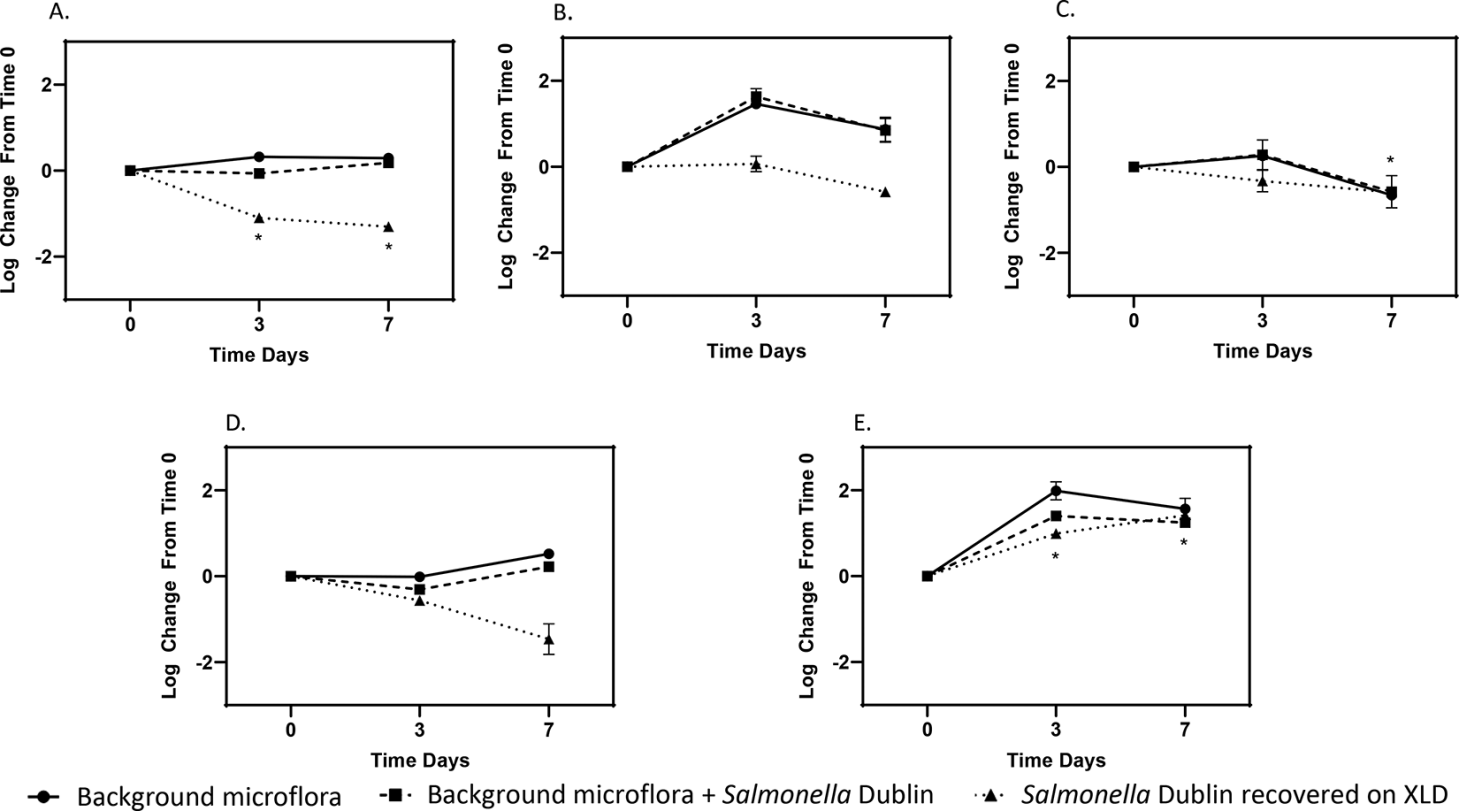
Survival of *Salmonella enterica* sv. Dublin in recycled bedding sand or gray water with a native microflora. *Salmonella* Dublin was inoculated (10^5^ log_10_ cfu/g sand) into triplicate tubes containing (A) sand collected from cow pens; (B) freshly recycled bedding sand; (C) bedding sand collected 4 d after recycling; (D) bedding sand collected 14 d after recycling; and (E) gray water from the recycling system. Results are expressed as the mean ± SD log_10_ cfu/g. Asterisks (*) represent a significant change (*P* < 0.05) in colony-forming units from time 0.

This study investigated the possibility that 3 bovine-associated serotypes of *Salmonella* can survive in sand used to bed dairy cows. Our initial experiments examined this question using sterile sand. The results of these experiments indicated all 3 serotypes of *Salmonella* persist in bedding sand for extended periods of time. The colony-forming units per gram levels in our experiments are within the predicted infectious dose for cattle, which is believed to follow ingestion of 10^4^ to 10^11^ cfu (Wray and Sojka, 1978; Wray and Davies, 2000; Ávila et al., 2011). In general, numbers of *Salmonella* in the sterile sand remained constant or increased slightly during the first 3 d, and then remained at that level or decreased somewhat through 28 d. There was some variation in the temporal response of the 3 serotypes. *Salmonella* sv. Cerro persisted the best of the 3 serotypes in sterile sand and was the only serotype for which we did not observe a subsequent decrease in colony-forming units per gram through 28 d (Figure 1A). We observed a moderate decrease in *Salmonella* sv. Dublin by d 14 (Figure 1C), and *Salmonella* sv. Heidelberg did not decrease until d 21 and 28 (Figure 1B.). Even during an extended 70-d period *Salmonella* sv. Dublin decreased less than 1 log_10_ cfu/g in sterile sand (Figure 1D).

The above experiments were performed using a relatively high inoculum of *Salmonella* (approximately 10^7^ to 10^8^ cfu/g sand). To understand how a lower inoculum would persist in sand, additional experiments were performed with *Salmonella* sv. Dublin. This serotype was selected because it is host-adapted and can become endemic on farms due to the bacterium's ability to asymptomatically infect cattle and be chronically shed in feces (Holschbach and Peek, 2018). In experiments with lower inoculation levels of *Salmonella* sv. Dublin (10^3^ and 10^5^ cfu/g) we unexpectedly observed up to a 3 log_10_ cfu/g increase in colony-forming units during the first 3 d (Figure 2). Although this is somewhat surprising because sand is assumed to be is a nutrient-poor environment (Godden et al., 2008; Justice-Allen et al., 2010), other investigators have made similar observations with a variety of bacterial species. Kristula et al. (2005) reported a significant increase in gram-negative bacteria and *Streptococcus* species during the first day of incubation in bedding sand, after which the bacterial populations remained steady for 7 d. Zdanowicz et al. (2004) similarly reported significant growth of coliforms and *Streptococcus* in bedding sand during the first day of incubation, after which the bacterial populations remained constant through the remainder of the study.

In addition to our findings with *Salmonella*, we observed a 3 to 4 log_10_ increase in colony-forming units in 48 h when *Escherichia coli* and *Enterococcus faecalis* were inoculated into sterile sand (data not shown). Analysis of bedding sand by other investigators found it contains approximately 0.03% nitrogen and 1.33% carbon (Justice-Allen et al., 2010). In our experiments the bedding sand was inoculated with a suspension of *Salmonella* in PBS and incubated at approximately 25°C. *Salmonella* is a mesophilic organism that grows best at temperatures of 20°C to 45°C, and readily replicates in environments in which there is a water activity of 0.93 or greater (Bolton et al., 1999). It appears that the remaining moisture from the PBS vehicle and nutrients in the sterile sand are sufficient for limited growth of *Salmonella* and other bacterial species.

Although *Salmonella* survived for extended periods of time in sterile sand, survival was diminished when *Salmonella* was inoculated into bedding sand with a complex microbial community. From prior work we know this sand has a diverse and robust microbial community (Pilch et al., 2021). Differences in the microbial communities were observed in sand at different stages in the recycling process, and in gray water used to wash the recycled sand (Pilch et al., 2021). In the present study we observed a significant decrease in *Salmonella* sv. Dublin colony-forming units on d 3 and 7 after it was inoculated into sand collected directly from freestalls in which cows were housed (Figure 3A). We also observed a decrease in *Salmonella* sv. Dublin after it was inoculated into recycled sand collected 4 or 14 d after washing (Figure 3C-D). Sand collected from the surface of the piles, or from a depth of 7 inches (17.78 cm) within the piles, exhibited similar inhibitory action against *Salmonella* sv. Dublin (data not shown). In contrast, we observed a significant increase in cfu of *Salmonella* sv. Dublin when inoculated into gray water from the sand recycling system (Figure 3E). Because gray water contains a high load of organic material and nutrients we assume competition for resources was reduced, allowing *Salmonella* to multiply.

Although our study did not directly address the impact of sand contamination on infection of cattle, clinically ill cattle can shed up to 10^9^ *Salmonella* per gram of feces, and up to 10^5^ organisms per mL of milk (McGuirk and Peek, 2003; Mohler et al., 2009). Because environmental contamination of *Salmonella* is a potential reservoir for infection of dairy cattle (Clegg et al., 1983; Vanselow et al., 2007), the results of our study suggest sand could be an environmental reservoir for *Salmonella*, albeit influenced by *Salmonella* serotype, availability of nutrients, and presence of competing microflora. Rodriguez et al. (2006) sampled bedding material, soil, and feed from dairy and beef cattle farms at 3-mo intervals during a 24-mo long study. *Salmonella* was routinely recovered from bedding, which was determined to be the most significant environmental reservoir. When Franz et al. (2005) introduced *Salmonella* sv. Typhimurium into cattle manure and soil fertilized with manure they found that *Salmonella* colony-forming units declined with time in the presence of the competitive microbiota. You et al. (2006) and Toth et al. (2011) both observed a decline in colony-forming units of *Salmonella* sv. Newport in bovine manure amended soils, although the organism persisted for extended periods of time (332 and 276 d, respectively). Our findings, together with these earlier reports, suggest that fecal shedding of *Salmonella* could result in contamination of bedding sand and serve as a potential source of infection for other cattle in the herd.
